# The Variability of the Order Burkholderiales Representatives in the Healthcare Units

**DOI:** 10.1155/2015/680210

**Published:** 2015-05-31

**Authors:** Olga L. Voronina, Marina S. Kunda, Natalia N. Ryzhova, Ekaterina I. Aksenova, Andrey N. Semenov, Anna V. Lasareva, Elena L. Amelina, Alexandr G. Chuchalin, Vladimir G. Lunin, Alexandr L. Gintsburg

**Affiliations:** ^1^N.F. Gamaleya Federal Research Center for Epidemiology and Microbiology, Ministry of Health of Russia, Gamaleya Street 18, 123098 Moscow, Russia; ^2^Federal State Budgetary Institution “Scientific Centre of Children Health” RAMS, 119991 Moscow, Russia; ^3^Research Institute of Pulmonology FMBA of Russia, 105077 Moscow, Russia

## Abstract

*Background and Aim*. The order Burkholderiales became more abundant in the healthcare units since the late 1970s; it is especially dangerous for intensive care unit patients and patients with chronic lung diseases. The goal of this investigation was to reveal the real variability of the order Burkholderiales representatives and to estimate their phylogenetic relationships. *Methods*. *16S rDNA* and genes of the *Burkholderia cenocepacia* complex (Bcc) Multi Locus Sequence Typing (MLST) scheme were used for the bacteria detection. *Results*. A huge diversity of genome size and organization was revealed in the order Burkholderiales that may prove the adaptability of this taxon's representatives. The following variability of the Burkholderiales in Russian healthcare units has been revealed: Burkholderiaceae (*Burkholderia*, *Pandoraea*, and *Lautropia*), Alcaligenaceae (*Achromobacter*), and Comamonadaceae (*Variovorax*). The *Burkholderia* genus was the most diverse and was represented by 5 species and 16 sequence types (ST). ST709 and 728 were transmissible and often encountered in cystic fibrosis patients and in hospitals. *A. xylosoxidans* was estimated by 15 genotypes. The strains of first and second ones were the most numerous. *Conclusions*. Phylogenetic position of the genus *Lautropia* with smaller genome is ambiguous. The Bcc MLST scheme is applicable for all Burkholderiales representatives for resolving the epidemiological problems.

## 1. Introduction

The antibiotic era resulted in the cardinal changes in the spectrum of the microorganisms, causing the healthcare-associated infections. Well-known bacterial pathogen* Staphylococcus aureus* was crowded by* Pseudomonas aeruginosa* [1]; then both were pressed by other Proteobacteria. The resistome of these bacteria has been enriched over the years of the nosocomial circulation, but most of them kept sensitive to at least one antibiotic.

The situation was complicated by the appearance of the order Burkholderiales bacteria after the late 1970s [2]. These bacteria are the common inhabitants of soil and water. They can be the plants' pathogens and have natural resistance to common antibiotics. They are especially dangerous for intensive care unit patients and patients with chronic lung diseases, particularly cystic fibrosis [3]. The taxonomy of this bacteria group has been developing since the 1980s and they were subdivided into different genera between 1981 and 2000 [4–8].

The infusion of nucleic acid sequencing technology in microbiology allowed Woese to start solving the bacterial phylogeny problem [9]. Proteobacteria, the most abundant and diverse bacterial phyla, were subdivided into classes on the base of the 16S rRNA gene sequences. First of all, 8–12 nucleotide signature sequences whose characteristic is unique to the species of Beta- and Gammaproteobacteria were identified (AAAAACCUUACC for Betaproteobacteria; AAACUCAAAUG for Gammaproteobacteria) [10, 11]. So the taxon* Pseudomonas*, according to Woese, was actually a collection of at least five separate groups of bacteria [9]. It was subdivided into several genera, one of which was genus* Burkholderia* [5]. Later a new genus* Ralstonia* was separated from* Burkholderia* [7].* Lautropia *[6]and* Pandoraea* [8] have appeared in the last few years. However the diversity, clinical and epidemiological significance of these taxa bacteria needs in detailed study. Continuing the investigation of* Bcc* role in nosocomial infections and using Multilocus Sequence Typing (MLST) as successful methodology in the epidemiology [12], we attempted to understand the variability of Burkholderiales in healthcare units.

## 2. Materials and Methods

### 2.1. Materials

Biological samples used for sequencing data are divided into two parts. The first part predominantly represented by nosocomial strains and strains from cystic fibrosis (CF) patients was described in [13] in detail. The second part contained some strains and mainly specimens of human sputum and aspirates from more than 300 CF patients.

### 2.2. DNA Isolation

DNA for PCR analysis was extracted from the bacterial cultures as described previously [13]. DNA from sputum and aspirate was isolated according to the protocol of the Maxwell 16 Tissue DNA Purification Kit for Maxwell MDX Instrument (Promega).

### 2.3. Species Identification

Identification of species was performed by amplification and sequencing of 16S ribosomal RNA gene (*16S rDNA*) fragment with primers [14, 15].

### 2.4. MLST

For Multilocus Sequence Typing, a modified scheme that allows differentiating 19 species of the* Burkholderia cepacia* complex (*Bcc*) was used [16]. The scheme includes the following targets for amplification:* atpD*, a *β* chain of ATP synthase;* gltB*, a large subunit of glutamate synthase;* gyrB*, a B subunit of DNA gyrase;* recA*, recombinase A;* lepA*, a GTP binding protein;* phaC*, acetyl CoA reductase; and* trpB*, a B subunit of tryptophane synthase. For DNA amplification, the following reagents were used: hot rescue DNA pol 5 units/*μ*L, PCR buffer 10x (N.F. Gamaleya Institute for Epidemiology and Microbiology MoH), dNTP5 mM (Medigen), and primers (Evrogen). The modified amplification program was the same for all targets: 95°C—10 min (95°C—30 s, 63°C—40 s, 72°C—1 min) × 35, 72°C—5 min.

### 2.5. PCR Products Sequencing

PCR products were sequenced according to the protocol of BigDye Terminator 3.1 Cycle Sequencing kit for the Genetic Analyzer 3130 of Applied Biosystems/Hitachi. The electrophoretic DNA separation was performed in 50 cm capillaries with POP7 polymer.

### 2.6. Nucleotide Sequence Analysis

Analysis of sequences and alignment were made by the use of the program ClustalW2 [17]. Allele numbers for MLST genes were assigned with the help of the PubMLST website [18]. New alleles and STs were controlled and submitted by the curator of* Bcc* MLST database. Identification of* 16S rDNA* sequences was carried out by BLAST search.

### 2.7. Nucleotide Sequence Polymorphism

The numbers of nucleotide/amino acid differences per site between concatenated sequences of 17 Bcc STs were obtained by pairwise distance calculation. Analyses were conducted in MEGA 4.0 [19].

Percent similarity and divergence coefficients of* gltB* gene nucleotide/amino acid sequences among analyzed representatives of the Burkholderiales were performed by the use of ClustalW2 [17], MEGA 6.0 [20], and MegAlign 5.05. For comparative sequence analysis and phylogenetic reconstruction 10 extra* gltB* gene sequences of the Burkholderiales order representatives (*Ralstonia solanacearum, Ralstonia pickettii, Acidovorax citrulli, Variovorax paradoxus, Bordetella bronchiseptica, Bordetella pertussis, *and* Lautropia mirabilis*) were retrieved from GenBank database (Table 3). The* gltB* sequences of* Pseudomonas aeruginosa *have been used as outgroup taxon (Table 3).

### 2.8. Phylogenetic Analysis

Phylogenetic analysis of* Bcc* was performed based on allelic profile data of* Bcc* STs and translated concatenated sequences of seven MLST loci. Phylogenetic tree of analyzed representatives of Burkholderiales order was constructed by the use of* gltB* sequences.

Analysis of profile data of* Bcc* STs was conducted using the software packages SplitsTree [21].

The phylogenetic tree of 17* Bcc* STs based on translated concatenated sequences of seven MLST loci was obtained automatically by applying the neighbor-joining method [22]. The evolutionary distances between 17* Bcc* STs were computed using the *p*-distance method [23] and were evaluated through the units of the amino acid differences' number per site. Evolutionary analyses were conducted in MEGA 6.0 [20]. Bootstrap analyses were performed with 500 replicates.

Phylogenetic tree of analyzed representatives of the Burkholderiales order was constructed by the use of neighborhood-joining, maximum likelihood, and maximum parsimony methods.

Genetic distances between microorganisms were evaluated by the use of Tamura 3-parameter model [24], which was chosen as an optimal evolution distance model derived from Modeltest based on the Akaike information criterion [25]. The evolutionary history was inferred by using the maximum likelihood method based on the general time reversible model GTR+G. Initial trees for the heuristic search were obtained automatically by applying neighbor-joining and BioNJ algorithms to a matrix of pairwise distances estimated by the use of the maximum composite likelihood approach and then selecting the topology with superior log likelihood value. A discrete gamma distribution was used to model evolutionary rate differences among sites (+G, parameter = 0.8170). Maximum parsimony trees were constructed with an algorithm implemented in MEGA 6.0. Bootstrap analyses were performed with 1,000 replicates.

## 3. Results and Discussion

### 3.1. Common Characteristics of the Burkholderiales Genomes

The Burkholderiales is the dominating order among the *β*-Proteobacteria available genomes, covering six families: Alcaligenaceae, Burkholderiaceae, Comamonadaceae, Oxalobacteraceae, Ralstoniaceae, and Sutterellaceae [26]. Four of them, demonstrated in Table 1, are more vital for the healthcare units. In the context of genome size, the order Burkholderiales is extraordinarily various (see S1 in Supplementary Material available online at http://dx.doi.org/10.1155/2014/680210): from the smallest 0.070281 Mb of the Burkholderiales bacterium JGI 0001003-L21 (the rhizosphere and endosphere of* Arabidopsis thaliana*, INSDC AUNS00000000.1 [27]) to the biggest 11.2941 Mb of the* Burkholderia terrae* (the forest soil, INSDC AKAU00000000.1 [28]). The genomes of the Burkholderiales representatives are organized in different number of the chromosomes: 1, 2, or 3, without genome size correlation. So 7.35915 Mb genome of* Achromobacter xylosoxidans* has one chromosome, but 7.00881 Mb genome of* Burkholderia multivorans* has three chromosomes [27].

Analysis of the small genome group has demonstrated that all of them are the genomes of the host-restricted microbial symbionts: of plants, as abovementioned Burkholderiales bacterium JGI 0001003-L21 (INCDS AUNS00000000.1, genome size 0.07 Mb) [27], of sap-feeding insects, as* Candidatus Zinderia insecticola* (INCDS CP002161.1, genome size 0.208564 Mb) [29], or of human, as Burkholderiales bacterium1_1_47 (INCDS ADCQ00000000.1, genome size 2.61 Mb), isolated from feces in Human Microbiome Project [27].

But most of the bacteria of* Burkholderia cepacia* complex (*Bcc*) pathogenic for human keep big genome, providing for the genome plasticity and adaptability [30].

### 3.2. Bcc Diversity in the Healthcare Units of the Russian Federation

In our investigation of the microorganisms, causing the healthcare-associated infections, we drew attention to* Bcc* bacteria in departments both common and specialized for cystic fibrosis (CF) patients. Thirteen genotypes (sequence type, ST) were detected in the first phase of the analysis and nine of them (708, 709, 710, 711, 712, 714, 727, 728, and 729) were identified for the first time (Table 2). It was shown that strains causing nosocomial infections in most cases refer to genotypes 728 and 708. Genotype 709 detected in strains isolated from patients in seven federal regions of Russia should be recognized as epidemiologically significant for patients with cystic fibrosis [13].

The extension of the specimens' sampling in the second phase of the investigation demonstrated new* Bcc* genotypes in* B. cenocepacia* (ST862, 878) and* B. multivorans* species (ST835) and continued prevalence of the ST709. 79% of the CF patients, infected by* Bcc*, had ST709 strain. So, 16 STs were detected for* Bcc* isolated from patients in RF.

**Figure 1:**
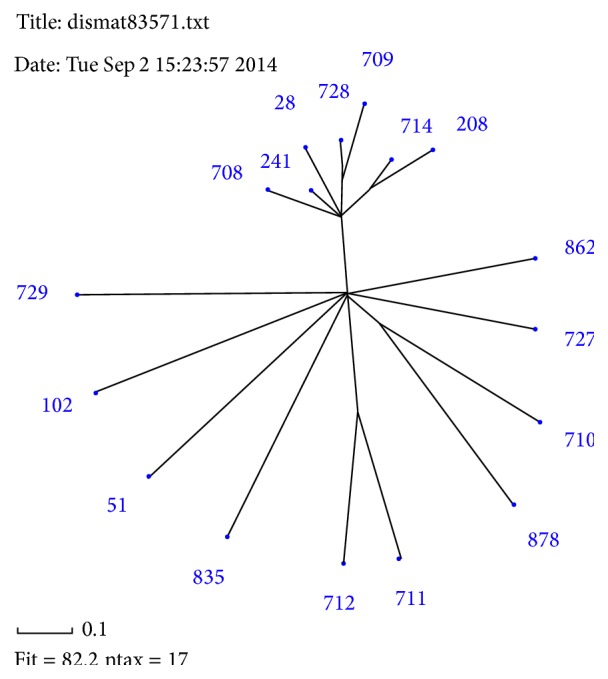
SplitsTree on the base of allelic profile data of the* Burkholderia cepacia* complex STs from RF.

To establish the relationship between different STs, we applied several methods of analysis. The first of them was SplitsTree analysis which was performed on the base of allelic profile data of* Bcc* STs (Figure 1). The most numerous group was formed by 6 STs of* B. cenocepacia* (708, 241, 728, 709, 714, and 208) closely related to the globally spread ST28. Next small groups were two STs containing first STs 710 and 878 that belonged to* B. cenocepacia* and second STs 711 and 712 related to* B. multivorans*. The other STs formed the separate branches.

**Figure 2:**
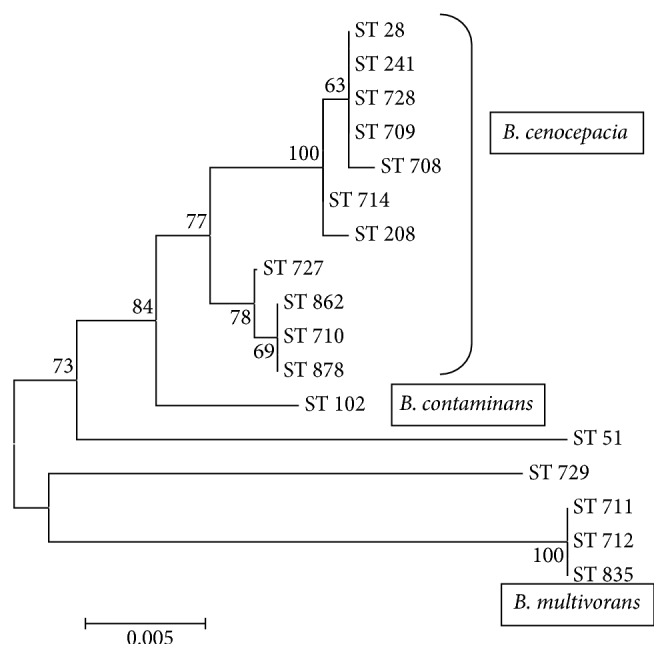
Neighbor-joining phylogenetic tree of seventeen* Burkholderia cepacia* complex STs based on translated concatenated sequences of seven MLST loci. ST51:* B. stabilis*; ST729:* B. vietnamiensis*.

To estimate the changes in the amino acid sequences, the concatenated sequences of MLST loci were translated. The bootstrap consensus tree using the neighbor-joining method was created (Figure 2). All groups of STs, represented different* Burkholderia* species, formed the separate branches with high bootstrap index (BI).

The most numerous group was formed by* B. cenocepacia*. It included 11 STs. Despite the fact that ST241, ST28, ST728, and ST709 had double locus variation (DLV) in the allelic profile, they formed the same branch with BI 63%. ST 708 which is also the DLV from ST241 was away from the group. This can be explained by amino acid residues changes in the translated MLST sequences. In fact, between ST28, ST241, ST709, and ST728 there were no amino acid residues replacements in translated sequences, while ST708 had replacement V82A in large subunit of glutamate synthase. STs 714 and 208 with DLV in their allelic profiles formed a separate subgroup (BI = 100%). These STs differed from the other STs with the replacement E52D in ATP synthase beta chain; ST208 had additional replacement E7D in acetoacetyl-CoA reductase.

Another subgroup of* B. cenocepacia* with BI = 79% was represented by strains with STs 727, 862, 710, and 878.

Evolutionary divergence between sequences of 17 STs was received by pairwise distance calculation (Supplementary Material, S2 and S3). The less variability (0.002–0.005) was within the group including ST28, ST241, ST728, ST709, ST714, ST208, and ST708 related to* B. cenocepacia* (group 1). Group 2 was formed by* B. cenocepacia* strains too (ST727, ST862, ST710, and ST878). They had 0.019–0.023 base differences per site in comparison with group 1. So, in whole intraspecies* B. cenocepacia* STs variability was 0.002–0.023. The* B. multivorans* STs variability was almost the same—0.003–0.011. The most closely related species in the analyzed sample of* Bcc* were* B. cenocepacia* and* B. contaminans *(ST102) with variability from 0.037 to 0.040.

However, in most cases nucleotide rearrangement did not lead to changes in amino acid residues sequences and polymorphism within amino acid sequences was less than within nucleotides (Supplementary Material, S2 and S3). ST28, ST241, ST728, and ST709 had the same amino acid residues sequences. ST714, ST208, and ST708 differed from them with 0.001–0.003 amino acid residues per site. So, the number of amino acid residues' differences per site in group 1 was 0.000–0.003. More detectable changes were between STs group 1 and strains from group 2 (ST727, ST862, ST710, and ST878), 0.007–0.010 amino acid residues per site.

So,* B. cenocepacia* ST241, ST728, ST709, ST714, ST208, and ST708 formed clonal complex, including ST28, which characterized the strains with global spread. Most of these STs were typical only for RF healthcare units (Table 2). Three STs (728, 708, and 709) were adaptive for epidemic spread.

### 3.3. The Potential of the Bcc MLST Scheme in the Burkholderiales Representatives Detection

During the second phase of the investigation we dealt not only with bacterial strains but also with a lot of samples of the sputum and aspirate. The* Bcc* MLST scheme adaptation to new conditions, amplification* Bcc* DNA in total DNA of the sample, suggested the apprehension of Spilker et al. [16] that degenerate primers, which allowed expansion of the modified* Bcc* MLST scheme, would not be specific only for* Burkholderia* species. First representative of Burkholderiales was* Achromobacter xylosoxidans*, in which* gltB* gene was amplified with the* Bcc* MLST scheme primers. After including this sequence in the analysis of the samples, we identified two different* gltB* alleles for this bacterium from the CF patients [13]. Then another thirteen* gltB* alleles were detected for* A. xylosoxidans*. The data analysis demonstrated the prevalence of the allele 1 and allele 2 among the CF patients from all federal regions of RF, except Far East, where only allele 3 was registered for* A. xylosoxidans*.

The increase of the number of* A. xylosoxidans* cases in the healthcare units, not only in CF patients, is according to the data of the new species registration. The data analysis of List of Prokaryotic Names with Standing in Nomenclature for the Burkholderiales order members demonstrated that over the last two years 18 new species of the* Burkholderia* genus have been registered [31], but only one was isolated from the human respiratory sample and others were environmental. On the other hand, species number of the* Achromobacter* genus increased two times during this period. All eight new species were clinical [32]. This data suggested* Achromobacter* significance as nosocomial bacterium.

Two targets of* Pandoraea pnomenusa* (*recA* and* gltB*) were amplified with the primers of* Bcc* MLST scheme too. But this dangerous and transmissible bacterium was isolated only from one CF patient. Some cases of* Lautropia mirabilis* were registered within one period of time. Only* gltB* gene was detectable by* Bcc* MLST primers. At last* Variovorax paradoxus*, detected in the group of the patients' samples, was amplified with* gyrB* primers. Detection of the seldom trace amount of* Ralstonia *spp. was possible with the* gltB* primers too.

So, we may conclude that the* Bcc* MLST scheme* gltB* primers are universal for most of the clinically significant Burkholderiales. The* gltB* gene sequences from our investigation and sequences avoided from GenBank were analyzed in the next step.* Bordetella* genus sequence included was explained by the importance of this genus as causative agent of human diseases.

### 3.4. The **g** **l** **t** **B** Gene Sequence Polymorphism

38 representatives of the order Burkholderiales and two of* Pseudomonas aeruginosa *(as an outgroup taxon) were used in analysis. A 414-base-pair alignment for the* gltB* gene region was obtained.

Totally 295 variable nucleotide sites have been detected; 240 of them characterized diversity of the order Burkholderiales analyzed representatives. Differences between representatives of the Burkholderiales and the outgroup taxa,* Pseudomonas aeruginosa,* reached 48.8% (Pse-Ral, Pse-Var-Aci); see Table 4. The differences among the investigated Burkholderiales bacteria varied from 0.2% to 32.5%. The* gltB *allele diversity in* Bcc* represented in analysis by five species was comparable to the diversity among* A. xylosoxidans* alleles and reached 6.8% and 5.1%, respectively (Supplementary Material, S4). These data suggested the close relatedness of the* Bcc* species. Percent of the differences in* gltB* gene sequence between representatives of the Alcaligenaceae family,* A. xylosoxidans *and* Bordetella bronchiseptica/Bordetella pertussis,* was 6.8–9.2%, indicating close relatedness of these taxa too. The* gltB* allele differences between other representatives of Burkholderiales fell into the range of 20–32.5%.

Surprisingly, the level of* gltB* gene sequence differences between the members of one family Burkholderiaceae (Bcc and* Lautropia mirabilis*) reached 27.9–31.6%, and that was more than differences between* Bcc* and the member of the other families: Ralstoniaceae (*Ralstonia*, 20.6–26.9%) and Comamonadaceae (*Acidovorax, Variovorax*, 25.2–28.6%) (Supplementary Material, S4). However, according to the data of Gerner-Smidt, based on variability of* 16S rDNA* the differences between* Lautropia mirabilis *and* Burkholderia cepacia *were 7.7% [6], which can characterize the higher resolution features of* gltB* gene sequences.

Amino acid residues variability of the translated* gltB* gene fragments was also evaluated (Supplementary Material, S5). In the sequence, consisting of 136 amino acid residues, 111 residues were variable. So, out of 240 SNPs, characterizing the diversity of Burkholderiales, 81 SNPs resulted in amino acid residues substitutions. The interspecies diversity of* A. xylosoxidans* was characterized by six amino acid residues substitutions; the diversity of* Bcc*, by nine substitutions.

### 3.5. Phylogeny of the Analyzed Burkholderiales Representatives Based on **g** **l** **t** **B** Sequences

**Figure 3:**
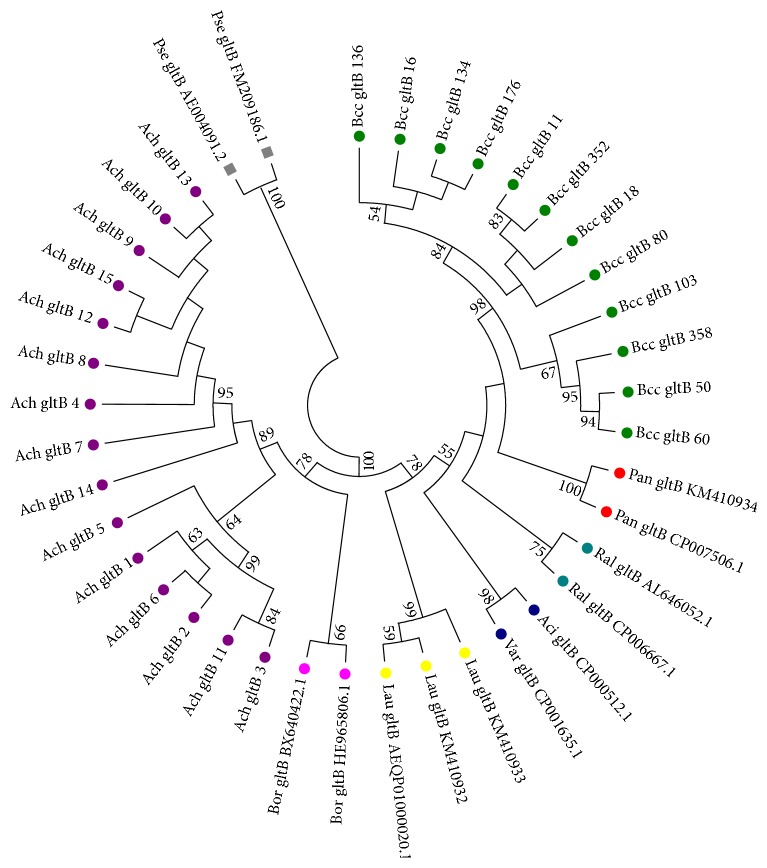
ML phylogenetic tree of analyzed representatives of Burkholderiales order based on* gltB* sequences.

ML phylogenetic tree based on* gltB *sequence is presented in Figure 3.* P. aeruginosa *well known as nosocomial bacterial pathogen, taken in this analysis as an outgroup taxon (Gammaproteobacteria, Pseudomonadales, and Pseudomonadaceae), formed the most divergent basal branch on the tree as was expected. The phylogenetic tree revealed two main groups of the Burkholderiales order representatives in this analysis. The first group (BI 78%) included only the members of the Alcaligenaceae family: fifteen alleles of* A. xylosoxidans* and reference* gltB* alleles from* Bordetella* genomes. It should be noted that* gltB* sequences allowed separating these taxa into distinct subclades.

The second group (BI 78%) was formed by the representatives of three families: Burkholderiaceae, Ralstoniaceae, and Comamonadaceae. Inside the second group twelve alleles of* Bcc* formed a large subgroup; two alleles of* Pandoraea pnomenusa *were closely related to this subgroup. However, three alleles of* Lautropia mirabilis* were more divergent from* gltB *alleles of Bcc and* Pandoraea pnomenusa* than representatives of two different families* Ralstoniacea* (*Ralstonia solanacearum, Ralstonia pickettii*) and* Alcaligenacea* (*Acidovorax citrulli, Variovorax paradoxus*).

A similar situation was described by phylogenetic cladogram, constructed for the order Burkholderiales representatives [33] by an automated pipeline of PATRIC genome database [34]. The construction of the phylogenetic tree on this server begins with amino acid sequence files for each genome. On this tree* Lautropia mirabilis *fell in one group (BI 79%) with two genomes of the host-restricted microbial symbionts:* Candidatus Zinderia insecticola* (INCDS CP002161.1) [29] and* Burkholderiales bacterium *1_1_47 (INCDS ADCQ00000000.1) [27], and with the representatives of the genera* Parasutterella* and* Sutterella*, the member of the family Sutterellaceae.* Parasutterella* was isolated from human faeces [35], and* Sutterella* strains were isolated from infections that occurred below the diaphragm [36]. Both have small genome 2.3769–2.98833 Mb.

*Lautropia mirabilis *is the poorly investigated species of a Gram-negative motile coccus with the unusual morphology fairly recently isolated from the human mouth by Gerner-Smidt et al. [6]. The genome of this bacterium is surprisingly small (3.15192 Mb) as compared with* Bcc* and* Pandoraea*. The loss of some genes in the evolution of this bacterium may be suggested.

On the other hand, the* 16S rDNA* sequencing data showed that* Lautropia mirabilis *belonged to a separate branch of the Betaproteobacteria and was most closely related to the genus* Burkholderia. *The isolated position together with the unique combination of chemotaxonomic and phenotypic properties allowed attributing of* Lautropia mirabilis *(strain AB2188) to the separate genus [6].

According to the last All-Species Living Tree (Release LTPs115, March 2014) [37] and also based on* 16S rDNA* sequences, single* Lautropia mirabilis *(AEQP01000026) formed a separate basal branch more related to* Burkholderia* and* Pandoraea* in Burkholderiaceae clade, which is joined to Comamonadaceae clade.

Similar disagreements between two phylogenetic trees were revealed for* Ralstonia* genus too.* Ralstonia* is usually attributed to Ralstoniaceae on the base of the* gltB* sequences, but according to All-Species Living Tree [37]* Ralstonia* is joined to* Cupriavidus* and fell into Oxalobacteraceae clade.

Consequently, according to our results, the polymorphism of* gltB* gene sequences was high and allowed describing substantial diversity of the Burkholderiales order members, defined the main taxonomical groups represented by Burkholderiaceae (*Burkholderia, Pandoraea, *and* Lautropia*), Alcaligenaceae (*Achromobacter*), and Comamonadaceae (*Variovorax*), and revealed significant differences between* Lautropia *and the other Burkholderiaceae taxa.

*In conclusion*, we identified and characterized quite a wide range of the Burkholderiales order bacteria which are vital for the healthcare units at present in Russia. They have been represented by five genera:* Burkholderia, Pandoraea, Lautropia* (Burkholderiaceae),* Achromobacter *(Alcaligenaceae), and* Variovorax* (Comamonadaceae). The most abundant were* Bcc* and* A. xylosoxidans* with prevalence of transmissible ST709 and ST728 of* Burkholderia cenocepacia* and the first and second genotypes of* A. xylosoxidans*. Also not common and unusual bacteria like* Pandoraea pnomenusa, Variovorax paradoxus, Lautropia mirabilis, *and* Ralstonia *spp. began to appear in the hospitals and were registered in the group of the patients' samples. These observations confirm profound changes in the spectrum of the microorganisms, causing the healthcare-associated infections over the past few years that can be associated with emergence and dissemination of novel antibiotic resistance from the natural reservoir to the clinical setting. So we may conclude that pathogenic potential of the Burkholderiales is on the increase. Clarification of some questions on bacteria phylogeny and future genomic analysis of Burkholderiales species will provide deeper large-scale insights into the evolution of virulence mechanisms. The timely identification of the Burkholderiales order representatives by genotyping is important to limit bacterial spread and so to resolve some epidemiological problems.

## Supplementary Material

S1. The genomes of the order Burkholderiales (taxid:80840) in the NCBI Genome List () 05-09-2014.
S2. Evolutionary divergence between nucleotide sequences of 17 STs.
S3. Evolutionary divergence between amino acid sequences of 17 STs.
S4. Percent similarity (upper triangle) and divergence coefficients (lower triangle) of gltB gene nucleotide sequences among analyzed representatives of Burkholderiales.
S5. Percent similarity (upper triangle) and divergence coefficients (lower triangle) of translated gltB amino-acid residues sequences among analyzed representatives of Burkholderiales.

## Figures and Tables

**Table 1:** The representatives of four families of the order Burkholderiales, which were detected in clinical specimens.

Class	Betaproteobacteria			
Order	Burkholderiales			
Family	Comamonadaceae	Alcaligenaceae	Burkholderiaceae	Ralstoniaceae
Genus	*Acidovorax *	*Bordetella *	*Burkholderia *	*Ralstonia *
Genus	*Variovorax *	*Achromobacter *	*Pandoraea *	
			*Lautropia *	

**Table 2:** The characteristics of the *Bcc* genotypes identified in RF.

Species	ST	ID PubMLST	Year	Source	Comments for ST distribution
*B. cenocepacia *	708	1149	2001	NON	Nosocomial strains in RF
*B. cenocepacia *	241	1258	2012	CF	Only CF strain in Far East of RF, but intercontinental spread strain in the world
*B. cenocepacia *	28	1268	1989	CF	Strains of multiple globally distinct locations, except for RF. Reference strain from Belgian collection
*B. cenocepacia *	728	1248	2004	NON	Nosocomial epidemic strains in RF, CF strain in all federal regions of RF
*B. cenocepacia *	709	1150	2008	CF	Epidemic strains for CF patients in all federal regions of RF, except for Far East
*B. cenocepacia *	714	1155	2003	NON	Strain from one hospital of the Southern Federal Region of RF
*B. cenocepacia *	208	1261	2012	CF	CF strains in Southern and Volga Federal Regions of RF and in USA
*B. cenocepacia *	862	1466	2014	CF	CF strain only in Far East of RF
*B. cenocepacia *	727	1246	2002	NON	Nosocomial strains in Northwestern Federal Region of RF
*B. cenocepacia *	710	1151	2012	CF	CF strains in RF
*B. cenocepacia *	878	1501	2014	CF	CF strain in RF
*B. multivorans *	711	1152	2012	CF	CF strains in RF
*B. multivorans *	712	1153	2011	CF	CF strains in RF and in Spain
*B. multivorans *	835	1443	2013	CF	CF strain in RF
*B. stabilis *	51	1267	1998	NON	Nosocomial strain in one hospital of RF, but intercontinental spread strain in the world
*B. contaminans *	102	1264	2000	CF	Nosocomial strain in one hospital and CF strain in Northwestern Federal Region of RF, but intercontinental spread strain in the world
*B. vietnamiensis *	729	1266	2012	CF	CF strain only in Far East of RF

CF: cystic fibrosis patient; NON: non-CF patient; RF: Russian Federation.

**Table 3:** The sources of the *gltB* gene sequences for the phylogenetic analysis; ^*∗*^consecutive laboratory numeration of the registered alleles.

*N*	Species	Source or GenBank accession number	*gltB* allele or locus tag
1	*B. cenocepacia *	[18]	11
2	*B. cenocepacia *	[18]	16
3	*B. stabilis *	[18]	18
4	*B. multivorans *	[18]	50
5	*B. multivorans *	[18]	60
6	*B. contaminans *	[18]	80
7	*B. vietnamiensis *	[18]	103
8	*B. cenocepacia *	[18]	134
9	*B. cenocepacia *	[18]	136
10	*B. cenocepacia *	[18]	176
11	*B. cenocepacia *	[18]	352
12	*B. multivorans *	[18]	358
13	*A. xylosoxidans *	KC817498	1^*∗*^
14	*A. xylosoxidans *	KC817500	2^*∗*^
15	*A. xylosoxidans *	KF290958	3^*∗*^
16	*A. xylosoxidans *	KF290959	4^*∗*^
17	*A. xylosoxidans *	KJ941209	5^*∗*^
18	*A. xylosoxidans *	KF297891	6^*∗*^
19	*A. xylosoxidans *	KF963246	7^*∗*^
20	*A. xylosoxidans *	KF963247	8^*∗*^
21	*A. xylosoxidans *	KF963248	9^*∗*^
22	*A. xylosoxidans *	KF963249	10^*∗*^
23	*A. xylosoxidans *	KF963250	11^*∗*^
24	*A. xylosoxidans *	KJ364657	12^*∗*^
25	*A. xylosoxidans *	KJ439616	13^*∗*^
26	*A. xylosoxidans *	KM262752	14^*∗*^
27	*A. xylosoxidans *	KM262753	15^*∗*^
28	*R. solanacearum *	AL646052.1	RSc2965
29	*R. pickettii *	CP006667.1	N234_19250
30	*A. citrulli *	CP000512.1	Aave_1008
31	*V. paradoxus *	CP001635.1	Vapar_1152
32	*B. bronchiseptica *	HE965806.1	BN112_3590
33	*B. pertussis *	BX640422.1	BP3753
34	*P. pnomenusa *	KM410934	KM410934
35	*P. pnomenusa *	CP007506.1	DA70_18115
36	*L. mirabilis *	KM410932	KM410932
37	*L. mirabilis *	KM410933	KM410933
38	*L. mirabilis *	AEQP01000020.1	EFV94423.1
39	*P. aeruginosa *	AE004091.2	PA5036
40	*P. aeruginosa *	FM209186.1	PLES_54261

**Table 4:** Percent of *gltB* sequences variability among analyzed representatives of Burkholderiales.

Group of genotypes	Variability, %	
	DNA sequence	Amino acid residues sequence
Lau	4.1–4.4	0.0–0.7
Ach	0.2–5.1	0.0–3.7
Bcc	0.2–6.8	0.0–5.1
Ach-Bor	6.8–9.2	5.9–8.1
Bcc-Pan	21.1–23.1	24.3–25.7
Bcc-Bor	25.5–26	33.1–36
Bcc-Ach	23.8–26.7	30.9–35.3
Bcc-Ral	20.6–26.9	25–30.9
Ach-Pan	25.5–27.2	25–27.9
Bor-Pan	26.9–27.4	27.9–29.4
Ral-Pan	20.9–27.7	24.3–27.2
Bcc-Var-Aci	25.2–28.6	29.4–38.2
Var-Pan	28.2–29.1	33.1–34.6
Bor-Lau	27.7–29.4	32.4–33.1
Var-Bor	27.9–29.4	35.3–39.7
Ral-Var	25.2–29.6	36–38.2
Ach-Var	27.4–29.6	35.3–41.9
Ach-Lau	28.2–30.3	32.4–35.3
Pan-Lau	30.1–30.6	30.9–31.6
Ral-Bor	24.5–31.3	30.9–36.8
Var-Lau	29.4–31.3	33.1–36
Ach-Ral	24–31.6	28.7–38.2
Bcc-Lau	27.9–31.6	30.1–33.1
Ral-Lau	26–32.5	30.9–34.6
Pse-Bor	42.7–43	66.2–66.9
Ach-Pse	43.7–45.9	66.2–66.9
Pse-Lau	46.1–46.8	69.1–69.9
Bcc-Pse	44.7–47.1	65.4–66.2
Pse-Pan	47.6–47.8	66.2
Pse-Ral	46.1–48.8	66.9–69.9
Pse-Var	47.3–48.8	66.2–67.6

Bcc: *Burkholderia cepacia* complex; Ach: *Achromobacter xylosoxidans*; Lau: *Lautropia mirabilis*; Ral: *Ralstonia solanacearum*/*Ralstonia pickettii*; Aci: *Acidovorax citrulli*; Var: *Variovorax paradoxus*, Bor: *Bordetella bronchiseptica*/*Bordetella pertussis*; Pan: *Pandoraea pnomenusa*; Pse: *Pseudomonas aeruginosa*.

## Abbreviations

MLST: Multilocus Sequence Typing
ST: Sequence type
DLV: Double locus variant
ML: Maximum likelihood
NJ: Neighbor-joining
CF: Cystic fibrosis and cystic fibrosis patient
NON: Non-CF patient
RF: Russian Federation
*Bcc*: * Burkholderia cepacia* complex
Ach: * Achromobacter xylosoxidans*
Lau: * Lautropia mirabilis*
Ral: * Ralstonia solanacearum/Ralstonia pickettii*
Aci: * Acidovorax citrulli*
Var: * Variovorax paradoxus*
Bor: * Bordetella bronchiseptica/Bordetella pertussis*
Pan: * Pandoraea pnomenusa*
Pse: * Pseudomonas aeruginosa.*
