# Supplementary material for: The Variability of the Order Burkholderiales Representatives in the Healthcare Units
Source: Biomed Res Int. 2015 May 31;2015:680210. doi: 10.1155/2015/680210 (PMC4465658; doi:10.1155/2015/680210)
Supplement: Supplementary file 1 — S1. The genomes of the order Burkholderiales (taxid:80840) in the NCBI Genome List () 05-09-2014. S2. Evolutionary divergence between nucleotide sequences of 17 STs. S3. Evolutionary divergence between amino acid sequences of 17 STs. S4. Percent similarity (upper triangle) and divergence coefficients (lower triangle) of gltB gene nucleotide sequences among analyzed representatives of Burkholderiales. S5. Percent similarity (upper triangle) and divergence coefficients (lower triangle) of translated gltB amino-acid residues sequences among analyzed representatives of Burkholderiales. [file 680210.f1.pdf]

S1. The genomes of the order Burkholderiales (taxid:80840) in the NCBI Genome List (http://www.ncbi.nlm.nih.gov/genome/browse/) 05-09-2014

N	Organism/Name	Kingdom	Group	SubGroup	Size (Mb)	Chr	Plasmids	Assemblies
1.	Burkholderiales bacterium JGI 0001003-L21	Bacteria	Proteobacteria	Betaproteobacteria	0,070281	-	-	1
2.	Comamonadaceae bacterium JGI 0001003-E14	Bacteria	Proteobacteria	Betaproteobacteria	0,113075	-	-	1
3.	Candidatus Zinderia insecticola	Bacteria	Proteobacteria	Betaproteobacteria	0,208564	1	-	1
4.	Comamonadaceae bacterium JGI 0001013-A16	Bacteria	Proteobacteria	Betaproteobacteria	0,295955	-	-	1
5.	Oxalobacteraceae bacterium JGI 0001004-J12	Bacteria	Proteobacteria	Betaproteobacteria	0,301938	-	-	1
6.	Oxalobacteraceae bacterium JGI 0001002-K6	Bacteria	Proteobacteria	Betaproteobacteria	0,368093	-	-	1
7.	Burkholderiales bacterium JGI 0001003-J08	Bacteria	Proteobacteria	Betaproteobacteria	0,394907	-	-	1
8.	Burkholderiales bacterium JGI 0001002-H06	Bacteria	Proteobacteria	Betaproteobacteria	0,415398	-	-	1
9.	Pelomonas	Bacteria	Proteobacteria	Betaproteobacteria	0,507844	-	-	2
10.	Leptothrix ochracea	Bacteria	Proteobacteria	Betaproteobacteria	0,510792	-	-	1
11.	Oxalobacteraceae bacterium JGI 0001012-C15	Bacteria	Proteobacteria	Betaproteobacteria	0,640975	-	-	1
12.	Burkholderiales bacterium JGI 0001003-A5	Bacteria	Proteobacteria	Betaproteobacteria	0,686115	-	-	1
13.	Oxalobacteraceae bacterium JGI 001010-B17	Bacteria	Proteobacteria	Betaproteobacteria	0,825556	-	-	1
14.	Rhizobacter	Bacteria	Proteobacteria	Betaproteobacteria	1,05799	-	-	2
15.	Taylorella asinigenitalis	Bacteria	Proteobacteria	Betaproteobacteria	1,63856	1	-	3
16.	Candidatus Glomeribacter gigasporarum	Bacteria	Proteobacteria	Betaproteobacteria	1,72695	-	-	1
17.	Taylorella equigenitalis	Bacteria	Proteobacteria	Betaproteobacteria	1,73212	1	-	3
18.	Brackiella oedipodis	Bacteria	Proteobacteria	Betaproteobacteria	1,9221	-	-	1
19.	Basilea psittacipulmonis DSM 24701	Bacteria	Proteobacteria	Betaproteobacteria	1,95907	1	-	1
20.	Polynucleobacter necessarius	Bacteria	Proteobacteria	Betaproteobacteria	2,15949	1	-	2
21.	Oligella urethralis	Bacteria	Proteobacteria	Betaproteobacteria	2,31161	-	-	1
22.	Sutterella parvirubra	Bacteria	Proteobacteria	Betaproteobacteria	2,3769	-	-	1
23.	Oxalobacter formigenes	Bacteria	Proteobacteria	Betaproteobacteria	2,48839	-	-	2

24.	Comamonadaceae bacterium H1	Bacteria	Proteobacteria	Betaproteobacteria	2,49225	-	1	1
25.	Brachymonas chironomi	Bacteria	Proteobacteria	Betaproteobacteria	2,50356	-	-	1
26.	Pelistega	Bacteria	Proteobacteria	Betaproteobacteria	2,50491	-	-	1
27.	Oxalobacteraceae bacterium JGI 0001004-K23	Bacteria	Proteobacteria	Betaproteobacteria	2,57348	-	-	1
28.	Glomeribacter sp. 1016415	Bacteria	Proteobacteria	Betaproteobacteria	2,63764	-	-	1
29.	Burkholderiales bacterium 1_1_47	Bacteria	Proteobacteria	Betaproteobacteria	2,65565	-	-	1
30.	Oligella ureolytica	Bacteria	Proteobacteria	Betaproteobacteria	2,6683	-	-	1
31.	Parasutterella excrementihominis	Bacteria	Proteobacteria	Betaproteobacteria	2,8317	-	-	1
32.	Sutterella wadsworthensis	Bacteria	Proteobacteria	Betaproteobacteria	2,98833	-	-	3
33.	Candidatus Symbiobacter mobilis CR	Bacteria	Proteobacteria	Betaproteobacteria	2,99184	1	-	1
34.	Alicycliphilus	Bacteria	Proteobacteria	Betaproteobacteria	3,00778	-	-	1
35.	Lautropia mirabilis	Bacteria	Proteobacteria	Betaproteobacteria	3,15192	-	-	1
36.	Herminiimonas	Bacteria	Proteobacteria	Betaproteobacteria	3,38088	-	-	1
37.	Limnobacter	Bacteria	Proteobacteria	Betaproteobacteria	3,39027	-	-	1
38.	Limnohabitans	Bacteria	Proteobacteria	Betaproteobacteria	3,41171	-	-	2
39.	Herminiimonas arsenicoxydans	Bacteria	Proteobacteria	Betaproteobacteria	3,42431	1	-	1
40.	Thiomonas intermedia	Bacteria	Proteobacteria	Betaproteobacteria	3,4621	1	2	1
41.	Comamonas granuli	Bacteria	Proteobacteria	Betaproteobacteria	3,50599	-	-	1
42.	Caldimonas manganoxidans	Bacteria	Proteobacteria	Betaproteobacteria	3,53212	-	-	1
43.	Simplicispira psychrophila	Bacteria	Proteobacteria	Betaproteobacteria	3,60077	-	-	1
44.	Comamonas badia	Bacteria	Proteobacteria	Betaproteobacteria	3,68102	-	-	1
45.	Bordetella avium	Bacteria	Proteobacteria	Betaproteobacteria	3,73225	1	-	1
46.	Burkholderia rhizoxinica	Bacteria	Proteobacteria	Betaproteobacteria	3,75014	1	2	1
47.	Bordetella holmesii	Bacteria	Proteobacteria	Betaproteobacteria	3,76689	1	-	19

48.	Thiomonas arsenitoxydans	Bacteria	Proteobacteria	Betaproteobacteria	3,78553	1	1	1
49.	Acidovorax ebreus	Bacteria	Proteobacteria	Betaproteobacteria	3,79657	1	-	1
50.	Hylemonella gracilis	Bacteria	Proteobacteria	Betaproteobacteria	3,82161	-	-	2
51.	Pusillimonas noertemannii	Bacteria	Proteobacteria	Betaproteobacteria	3,91698	-	-	1
52.	Pusillimonas	Bacteria	Proteobacteria	Betaproteobacteria	3,92481	1	1	1
53.	Castellaniella defragrans	Bacteria	Proteobacteria	Betaproteobacteria	3,95282	1	-	1
54.	Comamonas aquatica	Bacteria	Proteobacteria	Betaproteobacteria	3,95606	-	-	2
55.	Candidatus Burkholderia kirkii	Bacteria	Proteobacteria	Betaproteobacteria	4,00944	-	-	1
56.	Ramlibacter tataouinensis	Bacteria	Proteobacteria	Betaproteobacteria	4,07019	1	-	1
57.	Bordetella	Bacteria	Proteobacteria	Betaproteobacteria	4,07972	-	-	1
58.	Bordetella pertussis	Bacteria	Proteobacteria	Betaproteobacteria	4,15026	1	1	40
59.	Rubrivivax benzoatilyticus	Bacteria	Proteobacteria	Betaproteobacteria	4,16007	-	-	2
60.	Herbaspirillum massiliense	Bacteria	Proteobacteria	Betaproteobacteria	4,18827	-	-	1
61.	Alcaligenes faecalis	Bacteria	Proteobacteria	Betaproteobacteria	4,24831	-	-	3
62.	Rhodoferax saidenbachensis ED16	Bacteria	Proteobacteria	Betaproteobacteria	4,24939	-	-	1
63.	Thiomonas	Bacteria	Proteobacteria	Betaproteobacteria	4,38846	-	-	2
64.	Methylibium	Bacteria	Proteobacteria	Betaproteobacteria	4,44942	-	-	2
65.	Bordetella trematum	Bacteria	Proteobacteria	Betaproteobacteria	4,46463	-	-	1
66.	Oxalobacteraceae bacterium IMCC9480	Bacteria	Proteobacteria	Betaproteobacteria	4,5015	-	-	1
67.	Sphaerotilus natans	Bacteria	Proteobacteria	Betaproteobacteria	4,59282	-	-	1
68.	Comamonas composti	Bacteria	Proteobacteria	Betaproteobacteria	4,63407	-	-	1
69.	Methylibium petroleiphilum	Bacteria	Proteobacteria	Betaproteobacteria	4,64364	1	1	1
70.	Verminephrobacter aporrectodeae	Bacteria	Proteobacteria	Betaproteobacteria	4,6818	-	-	1
71.	Advenella mimigardefordensis	Bacteria	Proteobacteria	Betaproteobacteria	4,76413	1	1	1

72.	Acidovorax delafieldii	Bacteria	Proteobacteria	Betaproteobacteria	4,84209	-	-	1
73.	Advenella kashmirensis	Bacteria	Proteobacteria	Betaproteobacteria	4,84603	1	1	2
74.	Bordetella parapertussis	Bacteria	Proteobacteria	Betaproteobacteria	4,89957	1	1	2
75.	Comamonadaceae bacterium URHA0028	Bacteria	Proteobacteria	Betaproteobacteria	4,90412	-	-	1
76.	Herbaspirillum lusitanum	Bacteria	Proteobacteria	Betaproteobacteria	4,90487	-	-	1
77.	Leptothrix cholodnii	Bacteria	Proteobacteria	Betaproteobacteria	4,9094	1	-	1
78.	Ideonella	Bacteria	Proteobacteria	Betaproteobacteria	4,93675	-	-	1
79.	Rhodoferax ferrireducens	Bacteria	Proteobacteria	Betaproteobacteria	4,96978	1	1	1
80.	Rubrivivax gelatinosus	Bacteria	Proteobacteria	Betaproteobacteria	5,04325	1	-	2
81.	Alicycliphilus denitrificans	Bacteria	Proteobacteria	Betaproteobacteria	5,07075	1	2	2
82.	Hydrogenophaga	Bacteria	Proteobacteria	Betaproteobacteria	5,14453	-	-	2
83.	Derxia gummosa	Bacteria	Proteobacteria	Betaproteobacteria	5,18517	-	-	1
84.	Collimonas fungivorans	Bacteria	Proteobacteria	Betaproteobacteria	5,1869	1	1	1
85.	Comamonas	Bacteria	Proteobacteria	Betaproteobacteria	5,24376	-	1	1
86.	Polaromonas glacialis	Bacteria	Proteobacteria	Betaproteobacteria	5,28404	-	-	1
87.	Bordetella petrii	Bacteria	Proteobacteria	Betaproteobacteria	5,28795	1	-	3
88.	Hydrogenophaga intermedia	Bacteria	Proteobacteria	Betaproteobacteria	5,28813	-	-	1
89.	Massilia consociata	Bacteria	Proteobacteria	Betaproteobacteria	5,32616	-	-	1
90.	Acidovorax citrulli	Bacteria	Proteobacteria	Betaproteobacteria	5,35277	1	-	2
91.	Polaromonas naphthalenivorans	Bacteria	Proteobacteria	Betaproteobacteria	5,36614	1	8	1
92.	Herbaspirillum frisingense	Bacteria	Proteobacteria	Betaproteobacteria	5,40833	-	-	1
93.	Pandoraea pnomenusa	Bacteria	Proteobacteria	Betaproteobacteria	5,43513	1	-	2
94.	Acidovorax avenae	Bacteria	Proteobacteria	Betaproteobacteria	5,52228	1	-	2
95.	Acidovorax oryzae	Bacteria	Proteobacteria	Betaproteobacteria	5,52678	-	-	1

96.	Acidovorax radicis	Bacteria	Proteobacteria	Betaproteobacteria	5,53023	-	-	2
97.	Chitinimonas koreensis	Bacteria	Proteobacteria	Betaproteobacteria	5,5861	-	-	1
98.	Verminephrobacter eiseniae	Bacteria	Proteobacteria	Betaproteobacteria	5,59794	1	1	1
99.	Burkholderiales bacterium JOSHI_001	Bacteria	Proteobacteria	Betaproteobacteria	5,60063	1	-	1
100.	Herbaspirillum seropedicae	Bacteria	Proteobacteria	Betaproteobacteria	5,63315	1	-	2
101.	Pseudacidovorax intermedius	Bacteria	Proteobacteria	Betaproteobacteria	5,69128	-	-	1
102.	Pandoraea	Bacteria	Proteobacteria	Betaproteobacteria	5,77201	1	-	5
103.	Herbaspirillum huttiense	Bacteria	Proteobacteria	Betaproteobacteria	5,77792	-	-	1
104.	Massilia	Bacteria	Proteobacteria	Betaproteobacteria	5,79957	-	-	3
105.	Bordetella bronchiseptica	Bacteria	Proteobacteria	Betaproteobacteria	5,83141	1	-	62
106.	Polaromonas	Bacteria	Proteobacteria	Betaproteobacteria	5,89868	1	2	3
107.	Burkholderia mallei	Bacteria	Proteobacteria	Betaproteobacteria	5,93808	2	-	16
108.	Janthinobacterium agaricidamnosum	Bacteria	Proteobacteria	Betaproteobacteria	5,949	1	-	1
109.	Ralstonia solanacearum	Bacteria	Proteobacteria	Betaproteobacteria	5,96071	1	2	18
110.	Comamonas testosteroni	Bacteria	Proteobacteria	Betaproteobacteria	6,0627	1	4	7
111.	Pseudoduganella violaceinigra	Bacteria	Proteobacteria	Betaproteobacteria	6,11035	-	-	1
112.	Massilia alkalitolerans	Bacteria	Proteobacteria	Betaproteobacteria	6,11168	-	-	1
113.	Massilia timonae	Bacteria	Proteobacteria	Betaproteobacteria	6,1365	-	-	1
114.	Herbaspirillum rubrisubalbicans	Bacteria	Proteobacteria	Betaproteobacteria	6,15299	-	-	1
115.	Achromobacter arsenitoxydans	Bacteria	Proteobacteria	Betaproteobacteria	6,15666	-	-	1
116.	Burkholderia andropogonis	Bacteria	Proteobacteria	Betaproteobacteria	6,17866	-	-	1
117.	Janthinobacterium lividum	Bacteria	Proteobacteria	Betaproteobacteria	6,21274	-	-	2
118.	Duganella zoogloeoides	Bacteria	Proteobacteria	Betaproteobacteria	6,27295	-	-	1
119.	Herbaspirillum	Bacteria	Proteobacteria	Betaproteobacteria	6,28457	-	-	7

120.	Burkholderia dolosa	Bacteria	Proteobacteria	Betaproteobacteria	6,4204	3	-	2
121.	Burkholderia acidipaludis	Bacteria	Proteobacteria	Betaproteobacteria	6,47543	-	-	1
122.	Xenophilus azovorans	Bacteria	Proteobacteria	Betaproteobacteria	6,53517	-	-	1
123.	Burkholderia ginsengisoli	Bacteria	Proteobacteria	Betaproteobacteria	6,54189	-	-	1
124.	Massilia niastensis	Bacteria	Proteobacteria	Betaproteobacteria	6,59449	-	-	1
125.	Variovorax	Bacteria	Proteobacteria	Betaproteobacteria	6,63234	-	1	13
126.	Mitsuaria	Bacteria	Proteobacteria	Betaproteobacteria	6,65505	-	-	1
127.	Burkholderia grimmiae	Bacteria	Proteobacteria	Betaproteobacteria	6,7043	-	-	1
128.	Delftia tsuruhatensis	Bacteria	Proteobacteria	Betaproteobacteria	6,73215	-	-	1
129.	Curvibacter gracilis	Bacteria	Proteobacteria	Betaproteobacteria	6,74824	-	-	1
130.	Delftia	Bacteria	Proteobacteria	Betaproteobacteria	6,75271	1	1	4
131.	Ralstonia	Bacteria	Proteobacteria	Betaproteobacteria	6,76618	-	-	9
132.	Cupriavidus taiwanensis	Bacteria	Proteobacteria	Betaproteobacteria	6,77177	2	2	3
133.	Curvibacter lanceolatus	Bacteria	Proteobacteria	Betaproteobacteria	6,83252	-	-	1
134.	Achromobacter piechaudii	Bacteria	Proteobacteria	Betaproteobacteria	6,8922	-	-	2
135.	Burkholderia ubonensis	Bacteria	Proteobacteria	Betaproteobacteria	6,93253	-	-	1
136.	Delftia acidovorans	Bacteria	Proteobacteria	Betaproteobacteria	6,95318	1	4	4
137.	Burkholderia multivorans	Bacteria	Proteobacteria	Betaproteobacteria	7,00881	3	1	7
138.	Oxalobacteraceae bacterium AB_14	Bacteria	Proteobacteria	Betaproteobacteria	7,02801	-	-	1
139.	Burkholderia oklahomensis	Bacteria	Proteobacteria	Betaproteobacteria	7,04716	-	-	2
140.	Ottowia thiooxydans	Bacteria	Proteobacteria	Betaproteobacteria	7,04872	-	-	1
141.	Acidovorax	Bacteria	Proteobacteria	Betaproteobacteria	7,08623	1	2	7
142.	Janthinobacterium	Bacteria	Proteobacteria	Betaproteobacteria	7,11467	1	-	4
143.	Burkholderia kururiensis	Bacteria	Proteobacteria	Betaproteobacteria	7,12886	-	-	3

144.	Alcaligenes	Bacteria	Proteobacteria	Betaproteobacteria	7,13895	-	-	3
145.	Burkholderia thailandensis	Bacteria	Proteobacteria	Betaproteobacteria	7,24599	2	-	12
146.	Cupriavidus pinatubonensis	Bacteria	Proteobacteria	Betaproteobacteria	7,25529	2	2	1
147.	Burkholderia caledonica	Bacteria	Proteobacteria	Betaproteobacteria	7,28235	-	-	1
148.	Burkholderia glumae	Bacteria	Proteobacteria	Betaproteobacteria	7,28464	2	4	5
149.	Cupriavidus metallidurans	Bacteria	Proteobacteria	Betaproteobacteria	7,29187	1	3	5
150.	Achromobacter xylosoxidans	Bacteria	Proteobacteria	Betaproteobacteria	7,35915	1	2	5
151.	Burkholderia bryophila	Bacteria	Proteobacteria	Betaproteobacteria	7,38182	-	-	1
152.	Burkholderia pseudomallei	Bacteria	Proteobacteria	Betaproteobacteria	7,45365	2	1	68
153.	Burkholderia graminis	Bacteria	Proteobacteria	Betaproteobacteria	7,47726	-	-	1
154.	Variovorax paradoxus	Bacteria	Proteobacteria	Betaproteobacteria	7,51589	2	-	5
155.	Burkholderia_dilworthii	Bacteria	Proteobacteria	Betaproteobacteria	7,67907	-	-	1
156.	Burkholderia sprentiae	Bacteria	Proteobacteria	Betaproteobacteria	7,76106	-	-	1
157.	Burkholderia zhejiangensis	Bacteria	Proteobacteria	Betaproteobacteria	7,76722	-	-	1
158.	Burkholderia phenoliruptrix	Bacteria	Proteobacteria	Betaproteobacteria	7,81103	2	1	2
159.	Burkholderia ambifaria	Bacteria	Proteobacteria	Betaproteobacteria	7,85547	3	1	4
160.	Cupriavidus	Bacteria	Proteobacteria	Betaproteobacteria	7,89301	-	-	9
161.	Burkholderia ferrariae	Bacteria	Proteobacteria	Betaproteobacteria	7,93864	-	-	1
162.	Burkholderia heleia	Bacteria	Proteobacteria	Betaproteobacteria	8,00747	-	-	1
163.	Burkholderia pyrrocinia	Bacteria	Proteobacteria	Betaproteobacteria	8,0497	-	-	2
164.	Burkholderia cenocepacia	Bacteria	Proteobacteria	Betaproteobacteria	8,05578	3	1	13
165.	Ralstonia pickettii	Bacteria	Proteobacteria	Betaproteobacteria	8,12585	3	3	7
166.	Burkholderia phytofirmans	Bacteria	Proteobacteria	Betaproteobacteria	8,21466	2	1	1
167.	Burkholderia vietnamiensis	Bacteria	Proteobacteria	Betaproteobacteria	8,39107	3	5	1

168.	Cupriavidus basilensis	Bacteria	Proteobacteria	Betaproteobacteria	8,54679	-	-	2
169.	Burkholderia glathei	Bacteria	Proteobacteria	Betaproteobacteria	8,63738	-	-	1
170.	Burkholderia mimosarum	Bacteria	Proteobacteria	Betaproteobacteria	8,64028	-	-	3
171.	Burkholderia bannensis	Bacteria	Proteobacteria	Betaproteobacteria	8,64877	-	-	1
172.	Burkholderia lata	Bacteria	Proteobacteria	Betaproteobacteria	8,67628	3	-	1
173.	Burkholderia phymatum	Bacteria	Proteobacteria	Betaproteobacteria	8,67656	2	2	1
174.	Burkholderia fungorum	Bacteria	Proteobacteria	Betaproteobacteria	8,69621	-	-	1
175.	Azohydromonas australica	Bacteria	Proteobacteria	Betaproteobacteria	8,78678	-	-	1
176.	Burkholderia cepacia	Bacteria	Proteobacteria	Betaproteobacteria	8,94713	2	3	5
177.	Burkholderia gladioli	Bacteria	Proteobacteria	Betaproteobacteria	9,0523	2	4	3
178.	Bordetella hinzii	Bacteria	Proteobacteria	Betaproteobacteria	9,13822	-	-	8
179.	Burkholderia caribensis	Bacteria	Proteobacteria	Betaproteobacteria	9,43603	-	-	1
180.	Burkholderia nodosa	Bacteria	Proteobacteria	Betaproteobacteria	9,62797	-	-	1
181.	Burkholderia xenovorans	Bacteria	Proteobacteria	Betaproteobacteria	9,73114	3	-	1
182.	Cupriavidus necator	Bacteria	Proteobacteria	Betaproteobacteria	9,74138	2	2	3
183.	Burkholderia sordidicola	Bacteria	Proteobacteria	Betaproteobacteria	10,2619	-	-	1
184.	Burkholderia oxyphila	Bacteria	Proteobacteria	Betaproteobacteria	10,6477	-	-	1
185.	Burkholderia	Bacteria	Proteobacteria	Betaproteobacteria	10,9184	3	3	39
186.	Burkholderia terrae	Bacteria	Proteobacteria	Betaproteobacteria	11,2941	-	-	2

S2. Evolutionary divergence between nucleotide sequences of 17 STs.
The number of base differences per site from analysis between nucleotide sequences is shown. All results are based on the pairwise analysis of 17 sequences

	ST_28	ST_241	ST_728	ST_709	ST_714	ST_208	ST_708	ST_727	ST_862	ST_710	ST_878	ST_711	ST_712	ST_835	ST_51	ST_102	ST_729
ST_28																	
ST_241	0.003																
ST_728	0.003	0.002															
ST_709	0.005	0.004	0.003														
ST_714	0.002	0.003	0.003	0.004													
ST_208	0.004	0.003	0.003	0.004	0.002												
ST_708	0.004	0.003	0.003	0.003	0.004	0.003											
ST_727	0.021	0.021	0.020	0.020	0.019	0.019	0.019										
ST_862	0.022	0.022	0.021	0.022	0.020	0.021	0.021	0.008									
ST_710	0.022	0.022	0.022	0.022	0.021	0.021	0.020	0.008	0.009								
ST_878	0.023	0.023	0.022	0.022	0.021	0.022	0.021	0.009	0.010	0.005							
ST_711	0.057	0.057	0.056	0.057	0.056	0.056	0.057	0.056	0.058	0.058	0.058						
ST_712	0.058	0.058	0.057	0.058	0.057	0.057	0.058	0.056	0.058	0.059	0.060	0.003					
ST_835	0.060	0.060	0.060	0.060	0.058	0.058	0.059	0.058	0.061	0.060	0.061	0.011	0.011				
ST_51	0.051	0.052	0.051	0.051	0.050	0.051	0.051	0.047	0.049	0.049	0.048	0.065	0.066	0.068			
ST_102	0.037	0.038	0.038	0.037	0.037	0.037	0.038	0.040	0.040	0.040	0.040	0.065	0.066	0.067	0.054		
ST_729	0.060	0.061	0.060	0.058	0.059	0.059	0.059	0.056	0.058	0.058	0.058	0.063	0.064	0.066	0.066	0.063	

S3. Evolutionary divergence between amino acid sequences of 17 STs.
The number of amino acid residues differences per site from analysis between sequences is shown. All results are based on the pairwise analysis of 17 sequences.

	ST_28	ST_241	ST_728	ST_709	ST_714	ST_208	ST_708	ST_727	ST_862	ST_710	ST_878	ST_711	ST_712	ST_835	ST_51	ST_102	ST_729
ST_28																	
ST_241	0.000																
ST_728	0.000	0.000															
ST_709	0.000	0.000	0.000														
ST_714	0.001	0.001	0.001	0.001													
ST_208	0.002	0.002	0.002	0.002	0.001												
ST_708	0.001	0.001	0.001	0.001	0.002	0.003											
ST_727	0.008	0.008	0.008	0.008	0.007	0.008	0.009										
ST_862	0.009	0.009	0.009	0.009	0.008	0.009	0.010	0.001									
ST_710	0.009	0.009	0.009	0.009	0.008	0.009	0.010	0.001	0.000								
ST_878	0.009	0.009	0.009	0.009	0.008	0.009	0.010	0.001	0.000	0.000							
ST_711	0.035	0.035	0.035	0.035	0.034	0.035	0.036	0.036	0.037	0.037	0.037						
ST_712	0.035	0.035	0.035	0.035	0.034	0.035	0.036	0.036	0.037	0.037	0.037	0.000					
ST_835	0.035	0.035	0.035	0.035	0.034	0.035	0.036	0.036	0.037	0.037	0.037	0.000	0.000				
ST_51	0.033	0.033	0.033	0.033	0.032	0.033	0.034	0.027	0.026	0.026	0.026	0.049	0.049	0.049			
ST_102	0.014	0.014	0.014	0.014	0.013	0.014	0.015	0.011	0.010	0.010	0.010	0.034	0.034	0.034	0.030		
ST_729	0.035	0.035	0.035	0.035	0.034	0.035	0.036	0.034	0.033	0.033	0.033	0.041	0.041	0.041	0.041	0.033	

S4. Percent similarity (upper triangle) and divergence coefficients (lower triangle) of gltB gene nucleotide sequences among analyzed representatives of Burkholderiales

		Bcc_gltB_11	***Bcc_gltB_16***	***Bcc_gltB_18***	***Bcc_gltB_50***	***Bcc_gltB_60***	***Bcc_gltB_80***	***Bcc_gltB_103***	***Bcc_gltB_134***	***Bcc_gltB_136***	***Bcc_gltB_176***	***Bcc_gltB_352***	***Bcc_gltB_358***	***Ach_gltB_1***	***Ach_gltB_2***	***Ach_gltB_3***	***Ach_gltB_4***	***Ach_gltB_5***	***Ach_gltB_6***	***Ach_gltB_7***	***Ach_gltB_8***	***Ach_gltB_9***	***Ach_gltB_10***	***Ach_gltB_11***	***Ach_gltB_12***	***Ach_gltB_13***	***Ach_gltB_14***	***Ach_gltB_15***	***Ral_gltB_AL646052***	***Ral_gltB_CP006667***	***Aci_gltB_CP000512***	***Var_gltB_CP001635***	***Bor_gltB_HE965806***	***Bor_gltB_BX640422***	***Pan_gltB_KM410934***	***Pan_gltB_CP007506***	***Lau_gltB_KM410932***	***Lau_gltB_KM410933***	***Lau_gltB_EFV94423***	***Pse_gltB_AE004091***	***Pse_gltB_FM209186***
		**1**	**2**	**3**	**4**	**5**	**6**	**7**	**8**	**9**	**10**	**11**	**12**	**13**	**14**	**15**	**16**	**17**	**18**	**19**	**20**	**21**	**22**	**23**	**24**	**25**	**26**	**27**	**28**	**29**	**30**	**31**	**32**	**33**	**34**	**35**	**36**	**37**	**38**	**39**	**40**
**1**	***Bcc_gltB_11***	***	99,0	98,8	94,4	94,7	99,0	95,4	98,5	98,5	98,8	99,8	94,2	74,8	74,8	74,0	74,5	75,2	74,5	74,8	74,0	75,0	74,5	74,3	74,8	74,5	75,0	74,8	78,9	74,8	72,1	74,3	74,3	74,5	77,9	77,7	70,6	70,6	69,7	54,9	54,6
**2**	***Bcc_gltB_16***	1,0	***	98,8	94,4	94,7	99,0	96,4	99,5	99,5	99,8	98,8	94,2	74,8	75,0	74,3	74,8	75,5	74,8	75,0	74,3	75,2	74,8	74,5	75,0	74,8	75,2	75,0	79,4	75,5	72,8	74,8	74,5	74,8	78,4	78,2	70,6	71,1	69,7	55,3	55,1
**3**	***Bcc_gltB_18***	1,3	1,3	***	94,4	94,7	98,3	95,9	98,3	98,3	98,5	98,5	94,2	75,2	75,5	74,8	75,7	76,0	75,2	76,0	75,2	76,2	75,7	75,0	76,0	75,7	75,7	76,0	79,1	75,0	72,3	74,5	75,2	75,5	78,9	78,6	70,4	70,4	69,4	55,1	54,9
**4**	***Bcc_gltB_50***	6,0	6,0	6,0	***	99,8	93,4	95,1	93,9	93,9	94,2	94,2	98,1	74,3	74,5	73,8	74,3	74,8	74,3	74,5	73,8	74,8	74,3	74,0	74,5	74,3	74,5	74,5	76,9	73,1	71,4	74,8	74,3	74,3	77,2	76,9	72,1	71,4	70,1	52,9	52,9
**5**	***Bcc_gltB_60***	5,7	5,7	5,7	0,3	***	93,7	95,4	94,2	94,2	94,4	94,4	98,3	74,5	74,8	74,0	74,5	75,0	74,5	74,8	74,0	75,0	74,5	74,3	74,8	74,5	74,8	74,8	77,2	73,1	71,6	74,5	74,5	74,5	77,4	77,2	71,8	71,6	70,4	53,2	53,2
**6**	***Bcc_gltB_80***	1,0	1,0	1,8	7,1	6,8	***	95,9	98,5	98,5	98,8	98,8	93,2	74,5	74,5	73,8	74,3	75,0	74,3	74,5	73,8	74,8	74,3	74,0	74,5	74,3	74,8	74,5	78,4	74,5	71,8	74,0	74,0	74,3	77,7	77,4	70,4	70,4	69,4	54,6	54,4
**7**	***Bcc_gltB_103***	4,9	3,8	4,4	5,2	4,9	4,4	***	95,9	95,9	96,1	95,1	94,2	73,8	74,0	73,3	74,3	74,8	73,8	74,5	73,8	74,8	74,3	73,5	74,5	74,3	74,5	74,5	78,6	74,3	72,3	74,5	74,8	75,0	78,2	77,9	69,7	69,9	68,4	54,9	54,6
**8**	***Bcc_gltB_134***	1,5	0,5	1,8	6,5	6,3	1,5	4,4	***	99,5	99,8	98,3	93,7	74,8	75,0	74,3	74,8	75,5	74,8	75,0	74,3	75,2	74,8	74,5	75,0	74,8	75,2	75,0	79,1	75,0	72,8	74,3	74,5	74,8	78,4	78,2	70,6	71,1	69,4	54,9	54,6
**9**	***Bcc_gltB_136***	1,5	0,5	1,8	6,5	6,3	1,5	4,4	0,5	***	99,3	98,3	93,7	74,8	75,0	74,3	74,8	75,5	74,8	75,0	74,3	75,2	74,8	74,5	75,0	74,8	75,2	75,0	79,1	75,0	72,3	74,3	74,5	74,8	78,4	78,2	70,9	71,4	69,9	55,3	55,1
**10**	***Bcc_gltB_176***	1,3	0,3	1,5	6,3	6	1,3	4,1	0,3	0,8	***	98,5	93,9	75,0	75,2	74,5	75,0	75,7	75,0	75,2	74,5	75,5	75,0	74,8	75,2	75,0	75,5	75,2	79,1	75,2	73,1	74,5	74,8	75,0	78,2	77,9	70,6	71,1	69,4	55,1	54,9
**11**	***Bcc_gltB_352***	0,3	1,3	1,5	6,3	6	1,3	5,2	1,8	1,8	1,5	***	93,9	74,5	74,5	73,8	74,3	75,0	74,3	74,5	73,8	74,8	74,3	74,0	74,5	74,3	74,8	74,5	78,6	74,5	72,3	74,5	74,0	74,3	77,7	77,4	70,4	70,4	69,4	54,6	54,4
**12**	***Bcc_gltB_358***	6,3	6,3	6,3	2	1,8	7,4	6,3	6,8	6,8	6,5	6,5	***	75,0	75,2	74,5	74,0	75,5	75,0	74,3	73,5	74,5	74,0	74,8	74,3	74,0	74,8	74,3	76,9	73,3	71,6	74,5	74,3	74,5	78,6	78,4	72,1	71,8	70,6	52,9	52,9
**13**	***Ach_gltB_1***	30,3	30,3	29,6	31,1	30,7	30,7	31,9	30,3	30,3	30,0	30,7	29,9	***	99,8	99,0	95,9	97,8	99,3	96,1	95,4	95,4	95,9	99,3	96,1	95,6	96,8	96,1	75,0	69,4	71,6	71,6	92,0	91,3	73,5	73,3	71,4	71,4	70,6	54,9	54,6

**14**	***Ach_gltB_2***	30,3	30,0	29,2	30,7	30,3	30,7	31,5	29,9	30,0	29,6	30,7	29,5	0,3	***	99,3	96,1	98,1	99,5	96,4	95,6	95,6	96,1	99,5	96,4	95,9	97,1	96,4	75,2	69,7	71,8	71,8	92,2	91,5	73,8	73,5	71,1	71,1	70,4	54,6	54,4
**15**	***Ach_gltB_3***	31,4	31,1	30,3	31,8	31,5	31,8	32,6	31,1	31,1	30,7	31,8	30,7	1,0	0,8	***	95,4	97,3	98,8	95,6	94,9	94,9	95,4	99,8	95,6	95,1	96,4	95,6	74,8	69,4	71,4	71,1	92,2	90,8	73,8	73,5	70,4	70,6	69,7	54,1	53,9
**16**	***Ach_gltB_4***	30,7	30,3	28,8	31,0	30,7	31,0	31,1	30,3	30,3	29,9	31,0	31,4	4,4	4,2	5	***	97,6	95,6	99,3	99,0	98,5	99,0	95,6	99,3	98,8	97,6	98,8	75,2	68,9	71,4	70,9	93,0	93,0	73,8	73,5	71,4	71,1	70,9	56,3	56,1
**17**	***Ach_gltB_5***	29,6	29,2	28,5	30,3	29,9	29,9	30,3	29,2	29,2	28,8	29,9	29,2	2,3	2,1	2,9	2,6	***	97,6	97,8	97,1	97,1	97,6	97,6	97,8	97,3	97,6	97,8	76,0	69,9	72,6	72,3	93,2	92,7	74,5	74,3	70,9	70,9	70,1	55,8	55,6
**18**	***Ach_gltB_6***	30,7	30,3	29,6	31,1	30,7	31,1	31,9	30,3	30,3	30,0	31,1	29,9	0,8	0,5	1,3	4,7	2,6	***	95,9	95,1	95,1	95,6	99,0	95,9	95,4	96,6	95,9	75,0	69,2	71,4	71,4	91,7	91,0	73,3	73,1	70,6	70,6	69,9	54,6	54,4
**19**	***Ach_gltB_7***	30,3	29,9	28,5	30,7	30,3	30,7	30,7	29,9	29,9	29,6	30,7	31,1	4,2	3,9	4,7	0,8	2,3	4,4	***	99,3	98,8	99,3	95,9	99,0	99,0	97,8	99,5	75,5	69,2	71,1	71,1	93,2	93,2	73,5	73,3	71,1	71,8	70,6	55,8	55,6
**20**	***Ach_gltB_8***	31,4	31,0	29,6	31,8	31,4	31,8	31,8	31,0	31,0	30,7	31,8	32,2	5,0	4,7	5,5	1	3,1	5,3	0,8	***	99,0	99,5	95,1	99,3	99,3	97,1	99,3	74,8	68,4	70,9	70,4	92,5	92,5	73,3	73,1	70,9	71,1	70,4	55,6	55,3
**21**	***Ach_gltB_9***	29,9	29,6	28,1	30,3	29,9	30,3	30,3	29,5	29,6	29,2	30,3	30,7	5,0	4,7	5,5	1,5	3,1	5,3	1,3	1	***	99,5	95,1	99,3	99,3	97,1	99,3	75,5	69,4	70,9	71,1	92,5	92,5	73,8	73,5	71,8	71,8	71,1	56,3	56,1
**22**	***Ach_gltB_10***	30,7	30,3	28,8	31,0	30,7	31,0	31,1	30,3	30,3	29,9	31,0	31,4	4,4	4,2	5	1	2,6	4,7	0,8	0,5	0,5	***	95,6	99,8	99,8	97,6	99,8	75,0	68,9	71,4	70,9	93,0	93,0	73,3	73,1	71,4	71,4	70,6	56,1	55,8
**23**	***Ach_gltB_11***	31,0	30,7	29,9	31,4	31,1	31,4	32,2	30,7	30,7	30,3	31,4	30,3	0,8	0,5	0,3	4,7	2,6	1	4,5	5,3	5,3	4,7	***	95,9	95,4	96,6	95,9	74,8	69,4	71,4	71,4	92,2	91,0	73,8	73,5	70,6	70,6	69,9	54,4	54,1
**24**	***Ach_gltB_12***	30,3	29,9	28,5	30,7	30,3	30,7	30,7	29,9	29,9	29,6	30,7	31,1	4,2	3,9	4,70	0,80	2,30	4,40	1,00	0,80	0,80	0,30	4,50	***	99,5	97,8	99,5	75,2	69,2	71,6	71,1	93,2	93,2	73,5	73,3	71,6	71,1	70,9	56,3	56,1
**25**	***Ach_gltB_13***	30,7	30,3	28,8	31,0	30,7	31,0	31,1	30,3	30,3	29,9	31,0	31,4	4,7	4,5	5,30	1,30	2,80	5,00	1,00	0,80	0,80	0,30	5,00	0,5	***	97,3	99,5	74,8	68,7	71,1	70,9	92,7	93,0	73,1	72,8	71,4	71,4	70,6	56,1	55,8
**26**	***Ach_gltB_14***	29,9	29,6	28,8	30,7	30,3	30,3	30,7	29,6	29,6	29,2	30,3	30,3	3,4	3,1	3,90	2,60	2,60	3,60	2,30	3,10	3,10	2,60	3,70	2,3	2,9	***	97,8	75,0	69,9	71,4	70,9	92,5	92,2	73,3	73,1	71,1	71,6	70,9	55,8	55,6
**27**	***Ach_gltB_15***	30,3	29,9	28,5	30,7	30,3	30,7	30,7	29,9	29,9	29,6	30,7	31,1	4,2	3,9	4,7	1,3	2,3	4,4	0,5	0,8	0,8	0,3	4,5	0,5	0,5	2,3	***	75,2	69,2	71,1	71,1	93,2	93,2	73,1	72,8	71,1	71,6	70,4	55,8	55,6
**28**	***Ral_gltB_AL646052.1***	25,7	25,0	25,3	28,6	28,2	26,4	26,1	25,3	25,3	25,3	26,0	28,6	29,9	29,5	30,2	29,5	28,4	29,9	29,1	30,2	29,1	29,9	30,2	29,5	30,2	29,9	29,5	***	80,1	74,8	73,1	75,5	75,0	79,1	78,9	72,6	74,0	71,8	53,9	53,6
**29**	***Ral_gltB_CP006667.1***	27,5	26,4	27,1	30,1	30,1	27,8	28,2	27,1	27,1	26,8	27,8	29,7	34,2	33,8	34,1	35,0	33,4	34,6	34,6	35,8	34,2	35,0	34,2	34,6	35,4	33,4	34,6	19,9	***	72,8	70,4	68,7	68,9	72,6	72,3	68,0	68,9	67,5	51,2	51,0
**30**	***Aci_gltB_CP000512.1***	35,4	34,2	35,0	36,6	36,2	35,8	35,0	34,2	35,0	33,8	35,0	36,2	34,4	34,0	34,9	34,9	32,8	34,9	35,3	35,7	35,7	34,9	34,9	34,5	35,3	34,9	35,3	31,1	29,6	***	82,5	72,1	72,1	71,8	71,6	69,2	69,4	68,7	51,2	51,0
**31**	***Var_gltB_CP001635.1***	31,9	31,2	31,5	31,2	31,5	32,3	31,5	31,9	31,9	31,5	31,5	31,5	34,5	34,1	35,3	35,7	33,4	34,9	35,3	36,5	35,3	35,7	34,9	35,3	35,7	35,7	35,3	33,8	33,5	20,8	***	70,6	71,8	71,1	70,9	70,6	70,6	69,7	52,7	52,4

**32**	***Bor_gltB_HE965806.1***	31,0	30,7	29,5	31,0	30,7	31,4	30,3	30,6	30,7	30,3	31,4	31,0	8,9	8,6	8,6	7,8	7,5	9,2	7,5	8,3	8,3	7,8	8,6	7,5	8	8,3	7,5	29,1	35,4	33,6	36,1	***	96,6	73,1	72,8	71,4	72,3	70,6	57,0	56,8
**33**	***Bor_gltB_BX640422.1***	30,6	30,2	29,1	31,0	30,6	31,0	29,9	30,2	30,2	29,9	31,0	30,6	9,8	9,5	10,3	7,8	8,0	10,0	7,5	8,3	8,3	7,8	10,1	7,5	7,8	8,6	7,5	29,8	35,0	33,6	34,1	3,7	***	72,8	72,6	71,6	72,1	70,9	57,3	57,0
**34**	***Pan_gltB_KM410934***	27,2	26,4	25,7	28,2	27,9	27,6	26,8	26,4	26,4	26,8	27,6	26,1	32,1	31,8	31,8	31,8	30,6	32,5	32,2	32,5	31,8	32,5	31,8	32,2	32,9	32,5	32,9	25,3	30,8	35,9	37,0	32,9	33,3	***	99,8	69,9	69,7	69,9	52,4	52,7
**35**	***Pan_gltB_CP007506.1***	27,6	26,8	26,1	28,6	28,2	27,9	27,2	26,8	26,8	27,2	27,9	26,4	32,5	32,1	32,2	32,2	31,0	32,9	32,5	32,9	32,1	32,9	32,1	32,5	33,3	32,9	33,3	25,7	31,2	36,3	37,4	33,3	33,8	0,3	***	69,7	69,4	69,7	52,2	52,4
**36**	***Lau_gltB_KM410932***	38,7	38,7	39,2	36,3	36,7	39,2	40,4	38,7	38,3	38,7	39,2	36,3	35,7	36,2	37,4	35,7	36,6	37,0	36,1	36,6	34,9	35,7	37,0	35,3	35,7	36,1	36,1	35,6	38,4	40,4	37,9	35,8	35,4	40,0	40,4	***	95,9	95,6	53,9	53,9
**37**	***Lau_gltB_KM410933***	38,7	37,9	39,2	37,5	37,1	39,2	40,0	37,9	37,5	37,9	39,2	36,7	35,7	36,2	37,0	36,1	36,6	37,0	34,9	36,1	34,9	35,7	37,0	36,1	35,7	35,3	35,3	33,1	36,7	40,0	37,9	34,1	34,5	40,4	40,9	4,4	***	95,6	53,9	53,9
**38**	***Lau_gltB_EFV94423.1***	40,4	40,4	40,9	39,6	39,2	40,9	42,6	40,9	40,0	40,9	40,9	38,7	37,0	37,4	38,7	36,6	37,8	38,2	37,0	37,4	36,1	37,0	38,2	36,6	37,0	36,6	37,4	36,8	39,3	41,3	39,5	37,0	36,6	40,0	40,4	4,7	4,7	***	53,2	53,2
**39**	***Pse_gltB_AE004091.2***	70,0	68,8	69,5	75,4	74,7	70,6	69,9	70,0	68,8	69,5	70,6	75,3	67,7	68,4	69,7	64,3	65,6	68,3	65,4	66,0	64,2	64,8	69,0	64,3	64,8	65,3	65,4	72,7	74,9	79,2	74,8	62,3	61,8	76,3	77,0	72,2	72,2	74,2	***	99,8
**40**	***Pse_gltB_FM209186.1***	70,6	69,4	70,0	75,4	74,7	71,2	70,5	70,6	69,4	70,0	71,2	75,3	68,3	69,0	70,3	64,8	66,1	68,9	66,0	66,6	64,8	65,4	69,6	64,8	65,4	65,9	66,0	73,3	75,5	79,9	75,5	62,9	62,4	75,7	76,3	72,2	72,2	74,2	0,30	***

S5. Percent similarity (upper triangle) and divergence coefficients (lower triangle) of translated gltB amino-acid residues sequences among analyzed representatives of Burkholderiales

		Bcc_gltB_11	*Bcc_gltB_16*	*Bcc_gltB_18*	*Bcc_gltB_50*	*Bcc_gltB_60*	*Bcc_gltB_80*	*Bcc_gltB_103*	*Bcc_gltB_134*	*Bcc_gltB_136*	*Bcc_gltB_176*	*Bcc_gltB_352*	*Bcc_gltB_358*	*Ach_gltB_1*	*Ach_gltB_2*	*Ach_gltB_3*	*Ach_gltB_4*	*Ach_gltB_5*	*Ach_gltB_6*	*Ach_gltB_7*	*Ach_gltB_8*	*Ach_gltB_9*	*Ach_gltB_10*	*Ach_gltB_11*	*Ach_gltB_12*	*Ach_gltB_13*	*Ach_gltB_14*	*Ach_gltB_15*	*Ral_gltB_AL646052.1*	*Ral_gltB_CP006667.1*	*Aci_gltB_CP000512.1*	*Var_gltB_CP001635.1*	*Bor_gltB_HE965806.1*	*Bor_gltB_BX640422.1*	*Pan_gltB_KM410934*	*Pan_gltB_CP007506.1*	*Lau_gltB_KM410932*	*Lau_gltB_KM410933*	*Lau_gltB_EFV94423.1*	*Pse_gltB_AE004091.2*	*Pse_gltB_FM209186.1*
		**1**	**2**	**3**	**4**	**5**	**6**	**7**	**8**	**9**	**10**	**11**	**12**	**13**	**14**	**15**	**16**	**17**	**18**	**19**	**20**	**21**	**22**	**23**	**24**	**25**	**26**	**27**	**28**	**29**	**30**	**31**	**32**	**33**	**34**	**35**	**36**	**37**	**38**	**39**	**40**
**1**	*Bcc_gltB_11*	***	100	99,3	95,6	95,6	100	96,3	100	100	100	99,3	95,6	66,2	66,2	66,2	66,2	66,2	65,4	66,2	65,4	66,9	66,2	66,2	66,2	66,2	66,2	66,2	75	72,1	62,5	67,6	64,7	64,7	75	75	68,4	68,4	67,6	34,6	34,6
**2**	*Bcc_gltB_16*	0	***	99,3	95,6	95,6	100	96,3	100	100	100	99,3	95,6	66,2	66,2	66,2	66,2	66,2	65,4	66,2	65,4	66,9	66,2	66,2	66,2	66,2	66,2	66,2	75	72,1	62,5	67,6	64,7	64,7	75	75	68,4	68,4	67,6	34,6	34,6
**3**	*Bcc_gltB_18*	0,8	0,8	***	94,9	94,9	99,3	95,6	99,3	99,3	99,3	98,5	94,9	66,9	66,9	66,9	66,9	66,9	66,2	66,9	66,2	67,6	66,9	66,9	66,9	66,9	66,9	66,9	74,3	71,3	61,8	66,9	65,4	65,4	75,7	75,7	67,6	67,6	66,9	34,6	34,6
**4**	*Bcc_gltB_50*	4,7	4,7	5,5	***	100	95,6	97,1	95,6	95,6	95,6	94,9	100	68,4	68,4	68,4	68,4	68,4	67,6	68,4	67,6	69,1	68,4	68,4	68,4	68,4	68,4	68,4	72,8	69,1	62,5	70,6	66,2	66,9	75	75	69,9	69,9	69,1	33,8	33,8
**5**	*Bcc_gltB_60*	4,7	4,7	5,5	0	***	95,6	97,1	95,6	95,6	95,6	94,9	100	68,4	68,4	68,4	68,4	68,4	67,6	68,4	67,6	69,1	68,4	68,4	68,4	68,4	68,4	68,4	72,8	69,1	62,5	70,6	66,2	66,9	75	75	69,9	69,9	69,1	33,8	33,8
**6**	*Bcc_gltB_80*	0	0	0,8	4,7	4,7	***	96,3	100	100	100	99,3	95,6	66,2	66,2	66,2	66,2	66,2	65,4	66,2	65,4	66,9	66,2	66,2	66,2	66,2	66,2	66,2	75	72,1	62,5	67,6	64,7	64,7	75	75	68,4	68,4	67,6	34,6	34,6
**7**	*Bcc_gltB_103*	3,9	3,9	4,7	3,1	3,1	3,9	***	96,3	96,3	96,3	95,6	97,1	66,9	66,9	66,9	66,9	66,9	66,2	66,9	66,2	67,6	66,9	66,9	66,9	66,9	66,9	66,9	72,8	69,9	62,5	68,4	65,4	66,2	75,7	75,7	69,9	69,9	69,1	34,6	34,6
**8**	*Bcc_gltB_134*	0	0	0,8	4,7	4,7	0	3,9	***	100	100	99,3	95,6	66,2	66,2	66,2	66,2	66,2	65,4	66,2	65,4	66,9	66,2	66,2	66,2	66,2	66,2	66,2	75	72,1	62,5	67,6	64,7	64,7	75	75	68,4	68,4	67,6	34,6	34,6
**9**	*Bcc_gltB_136*	0	0	0,8	4,7	4,7	0	3,9	0	***	100	99,3	95,6	66,2	66,2	66,2	66,2	66,2	65,4	66,2	65,4	66,9	66,2	66,2	66,2	66,2	66,2	66,2	75	72,1	62,5	67,6	64,7	64,7	75	75	68,4	68,4	67,6	34,6	34,6
**10**	*Bcc_gltB_176*	0	0	0,8	4,7	4,7	0	3,9	0	0	***	99,3	95,6	66,2	66,2	66,2	66,2	66,2	65,4	66,2	65,4	66,9	66,2	66,2	66,2	66,2	66,2	66,2	75	72,1	62,5	67,6	64,7	64,7	75	75	68,4	68,4	67,6	34,6	34,6
**11**	*Bcc_gltB_352*	0,8	0,8	1,5	5,5	5,5	0,8	4,7	0,8	0,8	0,8	***	94,9	65,4	65,4	65,4	65,4	65,4	64,7	65,4	64,7	66,2	65,4	65,4	65,4	65,4	65,4	65,4	74,3	71,3	63,2	68,4	64	64	74,3	74,3	68,4	68,4	67,6	34,6	34,6
**12**	*Bcc_gltB_358*	4,7	4,7	5,5	0	0	4,7	3,1	4,7	4,7	4,7	5,5	***	68,4	68,4	68,4	68,4	68,4	67,6	68,4	67,6	69,1	68,4	68,4	68,4	68,4	68,4	68,4	72,8	69,1	62,5	70,6	66,2	66,9	75	75	69,9	69,9	69,1	33,8	33,8
**13**	*Ach_gltB_1*	44,8	44,8	43,5	40,8	40,8	44,8	43,5	44,8	44,8	44,8	46,2	40,8	***	100	100	98,5	98,5	98,5	98,5	97,8	97,8	98,5	100	98,5	98,5	99,3	98,5	70,6	63,2	59,6	64,7	93,4	93,4	73,5	73,5	66,9	66,9	66,2	33,1	33,1
**14**	*Ach_gltB_2*	44,8	44,8	43,5	40,8	40,8	44,8	43,5	44,8	44,8	44,8	46,2	40,8	0	***	100	98,5	98,5	98,5	98,5	97,8	97,8	98,5	100	98,5	98,5	99,3	98,5	70,6	63,2	59,6	64,7	93,4	93,4	73,5	73,5	66,9	66,9	66,2	33,1	33,1
**15**	*Ach_gltB_3*	44,8	44,8	43,5	40,8	40,8	44,8	43,5	44,8	44,8	44,8	46,2	40,8	0	0	***	98,5	98,5	98,5	98,5	97,8	97,8	98,5	100	98,5	98,5	99,3	98,5	70,6	63,2	59,6	64,7	93,4	93,4	73,5	73,5	66,9	66,9	66,2	33,1	33,1
**16**	*Ach_gltB_4*	44,8	44,8	43,5	40,8	40,8	44,8	43,5	44,8	44,8	44,8	46,2	40,8	1,6	1,6	1,6	***	100	97,1	100	99,3	99,3	100	98,5	100	100	99,3	100	70,6	63,2	59,6	64,7	94,1	94,1	74,3	74,3	66,9	66,9	66,2	33,8	33,8

17	***Ach_gltB_5***	44,8	44,8	43,5	40,8	40,8	44,8	43,5	44,8	44,8	44,8	46,2	40,8	1,6	1,6	1,6	0	***	97,1	100	99,3	99,3	100	98,5	100	100	99,3	100	70,6	63,2	59,6	64,7	94,1	94,1	74,3	74,3	66,9	66,9	66,2	33,8	33,8
18	***Ach_gltB_6***	46,2	46,2	44,8	42,2	42,2	46,2	44,8	46,2	46,2	46,2	47,6	42,2	1,6	1,6	1,6	3,1	3,1	***	97,1	96,3	96,3	97,1	98,5	97,1	97,1	97,8	97,1	69,9	61,8	58,1	63,2	91,9	91,9	72,1	72,1	65,4	65,4	64,7	33,1	33,1
19	***Ach_gltB_7***	44,8	44,8	43,5	40,8	40,8	44,8	43,5	44,8	44,8	44,8	46,2	40,8	1,6	1,6	1,6	0	0	3,1	***	99,3	99,3	100	98,5	100	100	99,3	100	70,6	63,2	59,6	64,7	94,1	94,1	74,3	74,3	66,9	66,9	66,2	33,8	33,8
20	***Ach_gltB_8***	46,2	46,2	44,8	42,2	42,2	46,2	44,8	46,2	46,2	46,2	47,6	42,2	2,3	2,3	2,3	0,8	0,8	4	0,8	***	98,5	99,3	97,8	99,3	99,3	98,5	99,3	69,9	62,5	59,6	64,7	93,4	93,4	73,5	73,5	66,2	66,2	65,4	33,1	33,1
21	***Ach_gltB_9***	43,5	43,5	42,2	39,5	39,5	43,5	42,2	43,5	43,5	43,5	44,8	39,5	2,3	2,3	2,3	0,8	0,8	4	0,8	1,6	***	99,3	97,8	99,3	99,3	98,5	99,3	71,3	64	58,8	64	93,4	93,4	75	75	67,6	67,6	66,9	33,1	33,1
22	***Ach_gltB_10***	44,8	44,8	43,5	40,8	40,8	44,8	43,5	44,8	44,8	44,8	46,2	40,8	1,6	1,6	1,6	0	0	3,1	0	0,8	0,8	***	98,5	100	100	99,3	100	70,6	63,2	59,6	64,7	94,1	94,1	74,3	74,3	66,9	66,9	66,2	33,8	33,8
23	***Ach_gltB_11***	44,8	44,8	43,5	40,8	40,8	44,8	43,5	44,8	44,8	44,8	46,2	40,8	0	0	0	1,6	1,6	1,6	1,6	2,3	2,3	1,6	***	98,5	98,5	99,3	98,5	70,6	63,2	59,6	64,7	93,4	93,4	73,5	73,5	66,9	66,9	66,2	33,1	33,1
24	***Ach_gltB_12***	44,8	44,8	43,5	40,8	40,8	44,8	43,5	44,8	44,8	44,8	46,2	40,8	1,6	1,6	1,6	0	0	3,1	0	0,8	0,8	0	1,6	***	100	99,3	100	70,6	63,2	59,6	64,7	94,1	94,1	74,3	74,3	66,9	66,9	66,2	33,8	33,8
25	***Ach_gltB_13***	44,8	44,8	43,5	40,8	40,8	44,8	43,5	44,8	44,8	44,8	46,2	40,8	1,6	1,6	1,6	0	0	3,1	0	0,8	0,8	0	1,6	0	***	99,3	100	70,6	63,2	59,6	64,7	94,1	94,1	74,3	74,3	66,9	66,9	66,2	33,8	33,8
26	***Ach_gltB_14***	44,8	44,8	43,5	40,8	40,8	44,8	43,5	44,8	44,8	44,8	46,2	40,8	0,8	0,8	0,8	0,8	0,8	2,3	0,8	1,6	1,6	0,8	0,8	0,8	0,8	***	99,3	71,3	63,2	59,6	64,7	93,4	93,4	74,3	74,3	66,9	66,9	66,2	33,8	33,8
27	***Ach_gltB_15***	44,8	44,8	43,5	40,8	40,8	44,8	43,5	44,8	44,8	44,8	46,2	40,8	1,6	1,6	1,6	0	0	3,1	0	0,8	0,8	0	1,6	0	0	0,8	***	70,6	63,2	59,6	64,7	94,1	94,1	74,3	74,3	66,9	66,9	66,2	33,8	33,8
28	***Ral_gltB_AL646052 .1***	31,6	31,6	32,7	35,1	35,1	31,6	35,1	31,6	31,6	31,6	32,7	35,1	37	37	37	37	37	38,3	37	38,3	35,8	37	37	37	37	35,8	37	***	77,9	61,8	63,2	69,1	69,1	75,7	75,7	69,1	69,1	68,4	33,1	33,1
29	***Ral_gltB_CP006667 .1***	31,6	31,6	32,7	36,3	36,3	31,6	35,1	31,6	31,6	31,6	32,7	36,3	44,8	44,8	44,8	44,8	44,8	47,6	44,8	46,2	43,5	44,8	44,8	44,8	44,8	44,8	44,8	22,9	***	64	62,5	63,2	63,2	72,8	72,8	66,2	66,2	65,4	30,1	30,1
30	***Aci_gltB_CP000512 .1***	52,9	52,9	54,4	52,9	52,9	52,9	52,9	52,9	52,9	52,9	51,4	52,9	57,2	57,2	57,2	57,2	57,2	60,4	57,2	57,2	58,8	57,2	57,2	57,2	57,2	57,2	57,2	54,4	44,4	***	81,6	60,3	61	65,4	65,4	64,7	64,7	64	32,4	32,4
31	***Var_gltB_CP00163 5.1***	43	43	44,4	37,9	37,9	43	41,7	43	43	43	41,7	37,9	46,7	46,7	46,7	46,7	46,7	49,6	46,7	46,7	48,1	46,7	46,7	46,7	46,7	46,7	46,7	51,4	47,1	22,1	***	63,2	64,7	66,9	66,9	66,9	66,9	66,2	33,8	33,8
32	***Bor_gltB_HE96580 6.1***	47,6	47,6	46,2	44,8	44,8	47,6	46,2	47,6	47,6	47,6	49,1	44,8	7,3	7,3	7,3	6,4	6,4	9	6,4	7,3	7,3	6,4	7,3	6,4	6,4	7,3	6,4	39,5	44,8	55,6	49,6	***	96,3	70,6	70,6	67,6	67,6	66,9	33,8	33,8
33	***Bor_gltB_BX64042 2.1***	47,6	47,6	46,2	43,5	43,5	47,6	44,8	47,6	47,6	47,6	49,1	43,5	7,3	7,3	7,3	6,4	6,4	9	6,4	7,3	7,3	6,4	7,3	6,4	6,4	7,3	6,4	39,5	44,8	54,1	46,7	4	***	72,1	72,1	67,6	67,6	66,9	33,1	33,1
34	***Pan_gltB_KM41093 4***	31,6	31,6	30,4	31,6	31,6	31,6	30,4	31,6	31,6	31,6	32,7	31,6	32,2	32,2	32,2	31	31	34,6	31	32,2	29,9	31	32,2	31	31	31	31	30,4	30,4	47,1	44,4	37	34,6	***	100	69,1	69,1	68,4	33,8	33,8
35	***Pan_gltB_CP00750 6.1***	31,6	31,6	30,4	31,6	31,6	31,6	30,4	31,6	31,6	31,6	32,7	31,6	32,2	32,2	32,2	31	31	34,6	31	32,2	29,9	31	32,2	31	31	31	31	30,4	30,4	47,1	44,4	37	34,6	0	***	69,1	69,1	68,4	33,8	33,8
36	***Lau_gltB_KM41093 2***	42,6	42,6	43,9	40	40	42,6	40	42,6	42,6	42,6	42,6	40	43,5	43,5	43,5	43,5	43,5	46,2	43,5	44,8	42,2	43,5	43,5	43,5	43,5	43,5	43,5	41,3	41,3	48,5	44,4	42,2	42,2	41,3	41,3	***	100	99,3	30,9	30,9
37	***Lau_gltB_KM41093 3***	42,6	42,6	43,9	40	40	42,6	40	42,6	42,6	42,6	42,6	40	43,5	43,5	43,5	43,5	43,5	46,2	43,5	44,8	42,2	43,5	43,5	43,5	43,5	43,5	43,5	41,3	41,3	48,5	44,4	42,2	42,2	41,3	41,3	0	***	99,3	30,9	30,9
38	***Lau_gltB_EFV9442 3.1***	43,9	43,9	45,3	41,3	41,3	43,9	41,3	43,9	43,9	43,9	43,9	41,3	44,8	44,8	44,8	44,8	44,8	47,6	44,8	46,2	43,5	44,8	44,8	44,8	44,8	44,8	44,8	42,6	42,6	50	45,7	43,5	43,5	42,6	42,6	0,8	0,8	***	30,1	30,1

**39**	***Pse_gltB_AE004091.2***	140	140	140	144	144	140	140	140	140	140	140	144	145	145	145	141	141	145	141	145	145	141	145	141	141	141	141	148	154	151	142	141	145	144	144	161	161	167	***	100
**40**	***Pse_gltB_FM209186.1***	140	140	140	144	144	140	140	140	140	140	140	144	145	145	145	141	141	145	141	145	145	141	145	141	141	141	141	148	154	151	142	141	145	144	144	161	161	167	0	***
